# Evaluation of Anaerobic Digestion Amended with Micro-Aeration and/or Sound Treatment on the Resistome and Virulence Factor Gene Profiles in Poultry Litter

**DOI:** 10.3390/antibiotics15020153

**Published:** 2026-02-02

**Authors:** Getahun E. Agga, John Loughrin

**Affiliations:** Food Animal Environmental Systems Research Unit, Agricultural Research Service, United States Department of Agriculture, Bowling Green, KY 42101, USA; john.loughrin@usda.gov

**Keywords:** anaerobic digestion, poultry litter, resistome, metagenomics, antimicrobial resistance, virulence factor genes

## Abstract

**Background**: Commercial broiler farms produce a large amount of litter that must be removed. Anaerobic digestion (AD) is animal manure management technology with the added benefit of producing reusable energy. Our team previously showed that the micro-aeration and sound treatment of animal manure during AD increase biogas production. However, their influence on antimicrobial resistance genes (ARGs) and bacterial virulence factor genes (VFGs) is unknown. Therefore, the objective of this study was to evaluate the effect of AD on the resistome and VFGs in poultry litter (PL) and see if the effect is modified by micro-aeration and/or sound treatments. **Methods**: A field experiment was conducted in four anaerobic digesters that consisted of a control (a standard AD system with no air or sound), micro-aeration, sound, and combined micro-aeration and sound treatments. Overall, 21 samples were collected and analyzed with shotgun metagenomic sequencing. The samples included digestate samples (n = 12) from the four digesters obtained at 6 (baseline, i.e., before beginning of micro-aeration and sound treatments), 23 and 42 weeks, raw PL samples (n = 4), two disks comprised of the same wood as the bedding material, an initial digestate seed sample, and two initial week 0 mix samples. **Results**: Across all sequence reads (n = 3190) obtained from 21 samples, over 80% of the resistome was composed of four antimicrobial classes: macrolides–lincosamides–streptogramins, tetracyclines, aminoglycosides, and glycopeptides. While the total number of ARGs declined in the control digestor, it increased over time in micro-aerated or sound-treated digesters, and their combination greatly increased the number of ARGs detected. This is a new finding, and it clearly shows that micro-aeration, sound, and their combination treatment during the anaerobic digestion of PL enriches ARGs. In contrast, sound-treated AD by itself significantly (*p* = 0.035) reduced the mean total ARG abundance compared to the control. The number and abundance of ARGs detected in the initial digestate and PL were lower than those in the AD samples, indicating their enrichment during the AD process. On the other hand, although the AD samples had a lower frequency and abundance of VFGs than the PL, AD did not completely remove the VFGs, and their detection frequency increased over time. While micro-aeration increased the abundance of VFGs compared to the control, this effect was countered by its combination with sound treatment, offering a good animal manure treatment strategy to reduce bacterial VFGs. **Conclusions**: Although additional research may be required, it was shown that while sound treatment may enrich the occurrence of ARGs, it seems promising to reduce the abundance of ARGs and VFGs during the AD of PL. On the other hand, micro-aeration, alone or when combined with sound treatment, increases the abundance of both ARGs and VFGs. Moreover, the study showed that AD, with or without micro-aeration and sound treatment, is not effective for the complete removal of ARGs and VFGs from poultry litter. Rather, AD systems may act as a hotspot for ARGs, and post-AD treatments such as composting need to be evaluated.

## 1. Introduction

Commercial broiler chicken farms produce a large amount of poultry litter (PL), and its large-scale accumulation on the farms is a major problem facing the poultry industry [1,2]. The land application of raw untreated PL, after a temporary storage in sheds at the broiler farms, is the primary means for its disposal to be used as organic fertilizer for crop production as a good source of nitrogen, phosphorus and potassium [2,3]. However, the potential risk for the spread of manure-borne pathogens including antimicrobial-resistant bacteria and their antimicrobial resistance genes (ARGs) from food animal production facilities into the environment is a major concern [3,4]. Current works by one of the authors showed that the impact of poultry litter in the soil is temporary, lasting at most one month, and its effect is more likely through nutrient provisions rather than the direct transfer of bacteria into the soil [5,6,7]. Animal manure management strategies such as lagoons, composting, and anaerobic digestion could play a crucial role in reducing antimicrobials, bacteria, and resistance genes transfer to the environment with differing effectiveness as previously reviewed [3,8].

Bacterial resistance to antimicrobials can be achieved through various biological mechanisms, which can be grouped into five broad categories [9,10,11]: (1) decreased accumulation of the antimicrobial within the cell, either through diminished permeability and/or active efflux of the antimicrobial from the bacterial cell; (2) inactivation of the antibiotics through the enzymatic modification or degradation of the antimicrobial; (3) target bypass by the acquisition of alternative metabolic pathways to those inhibited by the antimicrobial; (4) the modification or protection of the antimicrobial target; and (5) overproduction of the target enzyme.

**Figure 1:**
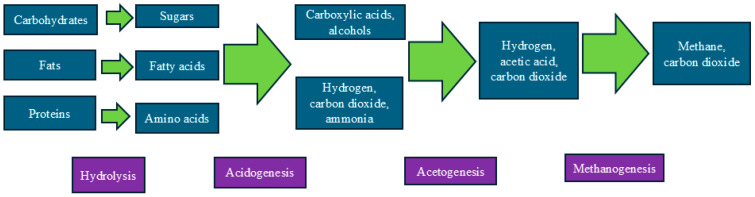
Schematic representation for microbiological mechanisms of anaerobic digestion of animal manure and poultry litter. The arrows show progressions of the four stages involved in anaerobic digestion system.

Anaerobic digestion (AD) is a microbial decomposition of organic matter in the absence of oxygen mainly mediated by methanogens primarily composed of archaea that are responsible for the production of methane (CH_4_), which is the main constituent of biogas [12]. Anaerobic digestion has an added benefit of producing renewable energy while reducing emissions from animal manure [3,12]. In addition, the digestate can be further composted or directly used as organic fertilizer [3,13]. AD can be conceptually separated into four concurrent stages consisting of hydrolysis, acidogenesis, acetogenesis, and methanogenesis (Figure 1), each of which involves cooperation between the specific microbial populations that exploit the metabolic end products of the other microorganisms [12,13].

The primary goal of AD is biogas production, which in so doing reduces the volume of the animal manure [8], and as such it was not designed as a means to remove antibiotics and antimicrobial resistance genes from manure [3]. Studies from our group evaluated the impact of conventional AD systems on tetracycline residues and tetracycline resistance genes with mixed results [8,14,15,16]. Although AD is strictly anaerobic, it was shown that micro-aeration increases biogas production [17]. In addition, previous studies showed increased biogas production due to sound treatment compared to control [17,18]. The effects were thought to be due to increased microbial activities through small amounts of air supplied and cavitation provided by the sound treatment that facilitate the degradation of the organic matter [17]. The microbial effect was shown to be through increased microbial diversity due to micro-aeration and sound treatments [19].

Although the effects of micro-aeration and sound treatment on biogas and microbiome composition were elucidated as described above [17,19], their effects on antimicrobial resistance and bacterial virulence factors have not been studied. Therefore, our objective was to study if AD systems supplemented with sound, micro-aeration, or both alter the resistome (total antimicrobial resistance genes) and virulence factor gene compositions of poultry litter beyond conventional AD systems.

## 2. Results

### 2.1. Description of Sequencing Outputs

The full resistome data are presented in Supplementary Table S1. Across all samples (n = 21), 3190 ARG accessions were obtained based on the origin of the bacterial genome to which the ARGs were aligned to. So, a specific ARG (e.g., *tet*(W) sequence reads could be detected more than once in a sample depending on the genome of the bacterial origin. Of the total ARG read counts, 1950 (61%) were from AD treatment groups with the experimental components yielding the remaining 1240 (39%) read counts (Table 1). While the proportions (22–27) of read counts were similar among the four AD treatment groups, among the experimental components, PL had the highest (39.4%) followed by wood disc (26.5%), initial mix (23.2%), and digestate (11%). The ARG reads were aligned to the genomes of 212 bacterial sources, including a few uncultured bacteria and plasmids. Only 5.3% of the ARG reads (n = 3190) were aligned to bacterial taxa profiles detected in the current samples. The genome alignments belonged to the following bacterial phyla: Firmicutes (43.6%), Proteobacteria (24.8%), Actinobacteria (10.7%), Bacteroidetes (5.3%), and Chlamydia (0.2%), and the remaining 15.4% represented unspecified bacterial sources (Supplementary Table S2).

Across all samples (n = 21), 337 unique genes and two unknown ARGs were detected from 3190 gene accessions (Supplementary Table S3), which were predominated by genes conferring resistance to antimicrobials (97%) with the remaining genes grouped into multidrug resistance (1.4%) or biocides (1.4%).

### 2.2. Effect of Anaerobic Digestion with or Without Micro-Aeration or Sound Treatment on Antimicrobial Resistance Genes

**Figure 2:**
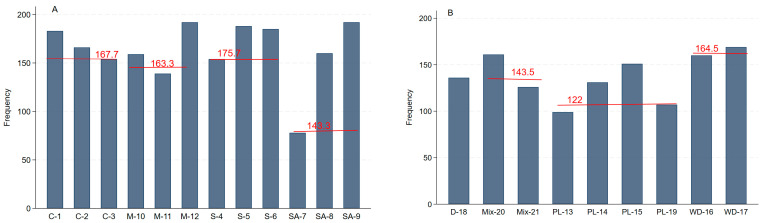
Total number of different antimicrobial resistance genes (ARGs) detected (**A**) by anaerobic digester treatment group; (**B**) by experimental component. The numbers on the top of the bar graphs show the mean number of ARGs detected within sample group. Sample keys: C-1 to C-3 indicate samples collected from control digesters at weeks 6, 23 and 42, respectively; M10-12: micro-aerated digester samples; S4-6: sound-treated digester samples; SA7-9: sound and micro-aerated digester samples. D-18: initial digestate samples used to seed the digesters; Mix20-21: mixture of initial digestate and poultry litter added to the digesters; PL13-19: samples from poultry litter that was added to the digesters at weeks 0, 6, 23, and 42; WD-16: wood disc obtained from control digesters; and WD-17: wood disc obtained from micro-aerated digesters.

The total number of ARGs detected is presented in Figure 2. The mean number of ARGs detected was not significantly (*p* > 0.05) affected by the treatment groups nor by the experimental components. Although the total number of ARGs showed a reduction in the control digester, it generally increased over time when AD was supplemented with micro-aeration and/or sound, and their combination greatly increased the number of ARGs detected (Figure 2A). The PL, on average, had fewer ARGs detected than the initial digestate and the initial mixes, while the wood discs had the most ARGs detected (Figure 2B).

**Figure 3:**
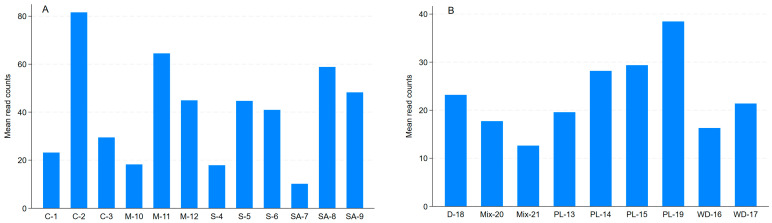
Mean total read counts in individual samples averaged over all antimicrobial resistance genes (ARGs) detected: (**A**) mean read counts of ARGs detected per sample averaged across all genes detected by treatment group; (**B**) mean read counts of ARGs detected per sample averaged across all genes detected in the experimental components. For sample keys, please refer to Figure 2.

**Figure 4:**
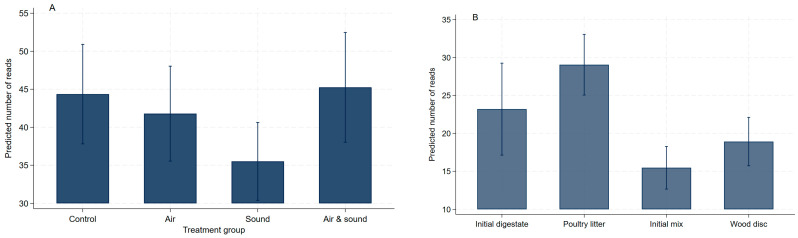
The effect of micro-aeration and sound treatments on the mean total abundance of antimicrobial resistance genes (**A**). The mean abundance of antimicrobial resistance genes in experimental components (**B**). For sample keys, please refer to Figure 2.

ARG read counts (i.e., gene abundance) increased by week 23 in all four treatment groups but greatly so in the control AD (Figure 3A). Based on individual samples, the PL feed generally had the highest mean read counts per sample, and the initial mixes (made up of the initial digestate seed from a commercial AD system and PL feed) had a low mean number of ARGs perhaps due to the dilution effect (Figure 3B). Averaged across the treatment groups, only sound-treated AD significantly (*p* = 0.035) reduced the mean total ARG abundance compared to the control AD with no effects of micro-aeration and the combination of micro-aeration and sound treatment (Figure 4A). The initial digestate and the PL used had significantly higher mean total ARG read counts than the initial mix and the wood discs (Figure 4B). This may be due to the dilution effect in the initial mixes. The PL provides the most abundant ARGs.

**Figure 5:**
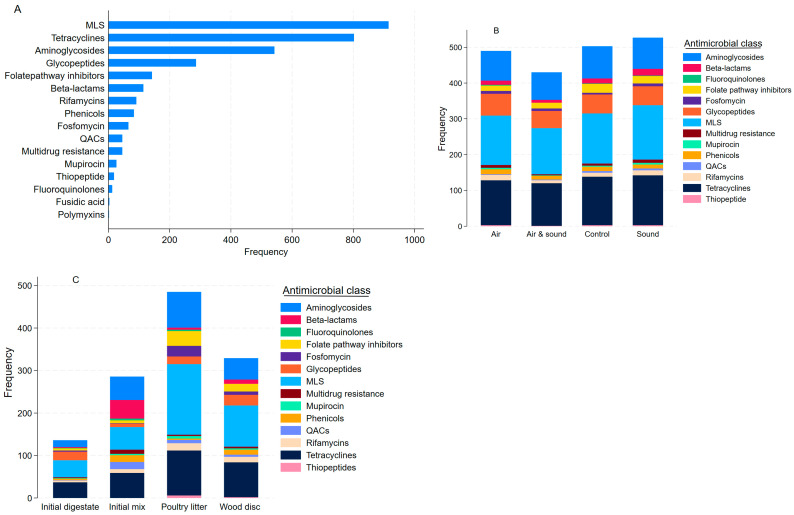
Frequency and composition of antimicrobial resistance genes detected by antimicrobial classes. Frequency of resistance genes aggregated by antimicrobial classes across all samples (**A**), antimicrobial class composition by treatment group (**B**), and by experimental components (**C**). MLS: macrolide–lincosamide–streptogramin resistance; QACs: quaternary ammonium compounds.

The top five ARGs across all sequence reads (n = 3190) were *tet*(W), *van*R, *mef*(A), *van*S and *tet*(M) (Supplementary Table S3). The ARGs belonged to 16 antimicrobial classes (Supplementary Table S3). Almost 80% of the resistome (n = 3190) belonged to four antimicrobial classes; macrolides–lincosamides–streptogramins (MLS; 29%), tetracyclines (25%), aminoglycosides (17%), and glycopeptides (9%), as shown in Supplementary Table S3 and Figure 5A. These four antimicrobial classes were dominant within each AD treatment with similar relative proportions (Figure 5B). However, some differences were observed among the experimental components (Figure 5C). In the initial digestate, the top four antimicrobial classes were MLS, tetracyclines, glycopeptides, and aminoglycosides, respectively; in the initial mix, they were tetracyclines, MLS, aminoglycosides, and beta-lactams, respectively; in the PL, the top four were MLS, tetracyclines, aminoglycosides, and folate pathway inhibitors, respectively; and in the wood disc, MLS, tetracyclines, aminoglycosides, and glycopeptides were the top classes, respectively. The ARG accessions (n = 3190) were grouped into 83 resistance mechanisms; the top six resistance mechanisms detected were tetracycline resistance ribosomal protection (13.5%), 23S rRNA methylation (11%), tetracycline efflux (10.3%), aminoglycoside nucleotidyl transferase (6.5%), aminoglycoside phosphorylation (6.3%), and lincosamide nucleotidyl transferase (6.2%), respectively, across all samples (Supplementary Table S3). These mechanisms were also the top six within each of the AD treatments. While most of these mechanisms were also among the top six within each of the experimental components, there were a few differences. The ABC-F-type ribosomal protection protein was among the top six resistance mechanisms in the initial digestate, poultry litter, and wood disc samples. In addition, the macrolide efflux major facilitator subfamily (MFS) transporter in the initial digestate samples and the class D beta-lactamase and quaternary ammonium compound efflux SMR transporter in the initial mix were among the top six mechanisms. The resistance mechanisms were generally grouped into five categories: drug inactivation (37.8%), efflux (24.4%), target protection (13.6%), target bypass (12.9%), and target modification (11.4%), respectively, across all samples (n = 21). Among the AD treatments, while this order was maintained in the sound-only-treated AD and AD treated with combined sound and micro-aeration, target bypass was ranked third in the control and micro-aerated AD. Except in the initial digestate samples (target bypass was 3rd), the ranking was maintained in other experimental components. In other words, drug inactivation and efflux were the most common resistance mechanisms in all samples.

#### 2.2.1. Macrolide, Lincosamide and Streptogramin (MLS) Resistance Genes

Across all samples (n = 21), 915 MLS gene accessions belonging to 65 gene groups and three resistance mechanisms were identified (Supplementary Table S4). The three mechanisms occurred almost at similar frequencies: target modification (36.3%), efflux (32.8%), and drug inactivation (30.9%).

Among the target-modifying genes, *erm* genes accounted for 92.8% (n = 332 accessions), with *erm* (C, F, G, X, B) representing the top five genes detected (Supplementary Table S4). Among the efflux protein-coding genes, *mef*(A) was the most prevalent, followed by *msr*(D) and *vga*(A) genes. These three gene groups together represented 81% of the total efflux coding MLS resistome. Among the genes coding for drug inactivation, *lnu*(A) was the most prevalent, remotely followed by *lnu*(B). The *lnu* genes together represented over 70% of the total drug-inactivating MLS resistome. Poultry litter had similar numbers of total ARG accessions detected except that poultry litter had higher drug-inactivating enzymes than each of the AD experiment groups. Of interest is the detection of *mph*(C) in the PL and not in AD samples, indicating its removal by the AD system; on the other hand, *lnu*(A) was the most prevalent in the PL and was also distributed among the ADs, indicating it did not change during AD.

Among the efflux-coding genes, *mef*(A), *vga*(A) and *msr*(D) that predominated the PL were also present in AD; on the other hand, *mef*(B), which was detected from AD, was not detected from the PL samples. Among the genes encoding for methylases, *erm* (38 and 46) was detected in the PL and was either not detected or detected rarely in AD; on the other hand, *erm* (A, B, D, F, G, 42, 47) detected in AD were not detected from the PL. However, *erm*(C) was the most prevalent in the PL and was detected at similar frequencies from the AD groups. The general trend is that some genes were detected in the PL and not in AD, showing an effective removal or reduction; some were detected among the AD processes but not in the PL, showing that these genes could originate from the digestate seed and survived the AD process; some were detected both in the PL and AD processes, showing no effect of the AD process. Some genes were detected from wood discs indicating their adsorbing ability.

#### 2.2.2. Tetracycline Resistance Genes

Overall, *tet*(W), *tet*(M), *tet*(L) and *tet*(O) were among the top prevalent genes detected both in the AD processes and the PL. While some genes such as *tet*(C), *tet*(H), *tet*(Q), and *tet*(32) were common in the AD treatments, they were absent or rarely detected from the PL. Since these genes were detected in the digestate seed and the wood discs, it can be hypothesized that the genes were introduced from the digestate and propagated during AD of the PL and adsorbed into the wood discs. Gene coding for tetracycline-inactivating protein, *tet*(X), was detected from micro-aeration and/or sound-treated AD groups as well as from the initial mixes and wood discs (Supplementary Table S4).

**Figure 6:**
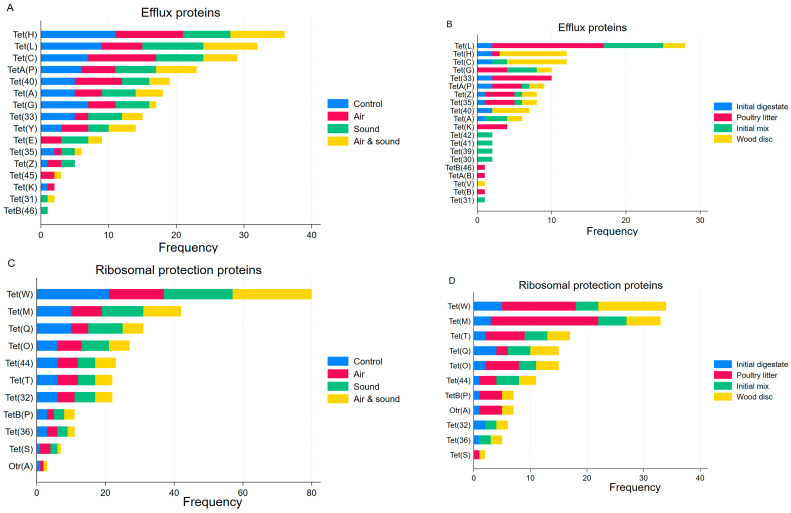
The distribution of tetracycline resistance genes by mechanisms and anaerobic digestion treatments and experimental components. Tetracycline resistance efflux proteins by anaerobic treatment (**A**) and by experimental components (**B**); tetracycline resistance ribosomal protection proteins by anaerobic digestion treatment (**C**) and by experimental components (**D**).

The number of genes coding for efflux and ribosomal protection proteins was found at similar frequencies between the PL and AD groups (Table 1). Among the efflux protein-coding genes, while *tet*(H) predominated the AD treatment groups, *tet*(L) was the most detected in the pre-digested PL (Figure 6). Among the genes coding for the ribosomal protection proteins, *tet*(W) and *tet*(M) were the most common both among the AD treatment groups and the AD components (Figure 6). The wood discs had the next higher number of *tet* genes detected, specifically *tet*(H) and *tet*(C), which were either non-detected or were infrequently detected in the PL but were among the top genes detected among the AD treatment groups, showing that the genes originated from the municipal digestate seed and that they were enriched during the AD process and adsorbed to the wood discs.

#### 2.2.3. Aminoglycoside Resistance

Aminoglycoside resistance almost exclusively consisted of genes coding for three types of aminoglycosides-modifying enzymes. Aminoglycoside acetyltransferases (AACs) represented 5.4% of the aminoglycoside resistance gene accessions detected among all the AD treatment groups compared with 17.6% in the experimental components. Aminoglycoside phosphotransferases (APHs) were detected in 40.8% of the total aminoglycoside resistome detected in AD groups and in 31.7% in the experimental components. Aminoglycoside nucleotidyltransferases (ANTs), including aminoglycoside adenyltransferases (AADs), were represented in 42.6% and 38.5% of the aminoglycoside resistome in the AD treatments and experimental groups, respectively (Supplementary Table S4). The total number of aminoglycoside resistance genes detected in pre-digestion PL was similar to the AD treatment groups (Table 1). However, more diverse aminoglycoside resistance genes (in terms of number) were detected in experimental constituents than the AD treatments. Similar total aminoglycoside resistance gene counts were detected between the PL and the AD treatment groups.

#### 2.2.4. Glycopeptide Resistance

The glycopeptide resistance genes detected in this study were primarily composed of vancomycin resistance genes (Supplementary Table S4). More diverse (i.e., more types of genes) glycopeptide resistance genes were detected in the AD treatment groups than in the experimental components, suggesting the enrichment of these genes during AD. About the same frequency of the glycopeptide resistance genes were detected in the digestate used as a seed and the PL feed—more genes and the highest overall frequency were detected in the wood discs (Table 1). The findings suggest that initial digestate from the commercial AD system and PL were the source but with different gene types. The *van*R and *van*S are the dominant glycopeptide genes in all sample types.

#### 2.2.5. Folate Pathway Inhibitor Resistance

The overall frequency of sulfonamide resistance genes detected in the pre-digested PL was higher than that in any of the AD treatment groups (Table 1). This again suggests that PL was the source of the genes detected in the other groups. Micro-aeration alone and micro-aeration and sound combination-treated AD groups had the lowest frequency of sulfonamide resistance genes. This shows the potential impact of the micro-aerated AD system in removing ARGs from animal manure. Two resistance gene families were identified: *dfr* genes, that confer resistance to the trimethoprim, occurred in ~32% of the total sulfonamide metagenomes both in the AD treatments and the experimental components. The remaining 68% of the sulfonamide resistome was comprised of *sul* genes, which confer resistance to sulfonamides. Among the *sul* genes, *sul*1 was the most prevalent in all sample types (Supplementary Table S4).

#### 2.2.6. Other Antimicrobial Resistance Gene Classes

Higher read counts of beta-lactam resistance genes were detected in experimental components than the AD groups (61 vs. 53 total frequencies, respectively) with more diverse genes being detected in experimental components than the AD groups (47 vs. 20 genes, respectively; Supplementary Table S4). Surprisingly, the initial mix had the greatest read counts of beta-lactam resistance genes (37 different genes with 44 total observations) than any group (Table 1). Plasmid-borne floroquinolone resistance gene *qep*A that encodes efflux pumps belonging to MSF were detected four times among the AD treatments and eight times among the experimental components (Table 1, Supplementary Table S4). The fosfomycin resistance genes detected belonged to two gene families *fos* and *fom*. Untreated PL had the highest frequency of fosfomycin resistance genes detected (Table 1, Supplementary Table S4). Phenicol resistance genes were detected at low frequencies in the commercial digestate seed and the PL feed, but they were enriched during mixing and AD processes (regardless of treatments) and adsorbed to wood discs (Table 1). Approximately 92% of the phenicol resistome was composed of *cat* genes in the AD experimental groups and 60% in the experimental components (Supplementary Table S4). The remaining 8% among the AD experimental treatments and 40% in the experimental components were composed of genes coding for the MFS efflux proteins *cmx*, *fex*A, *flo*R, and *cml*.

The MDR genes were detected at low frequencies in the commercial digestate seed and the PL; their frequencies in the initial mix were similar to those of micro-aerated AD and sound AD with the lowest occurrence being observed in combined micro-aeration and sound-treated AD (Table 1). The MDR genes were mainly made up of *oqx* group (Supplementary Table S4). Quaternary ammonium compounds were represented by 11 *qac* genes that were detected 45 times overall. Over two-thirds (69%) of the *qac* genes occurred in experimental components rather than in the AD groups (Table 1). The most frequently detected gene was *qac*EΔ1 (33.3%, n = 45), which was followed by *qac*F and *qac*L each occurring in 15.6% of the biocide resistome (Supplementary Table S4).

### 2.3. Effect of Anaerobic Digestion Treated with Micro-Aeration and/or Sound on Virulence Factor Genes in Poultry Litter

**Figure 7:**
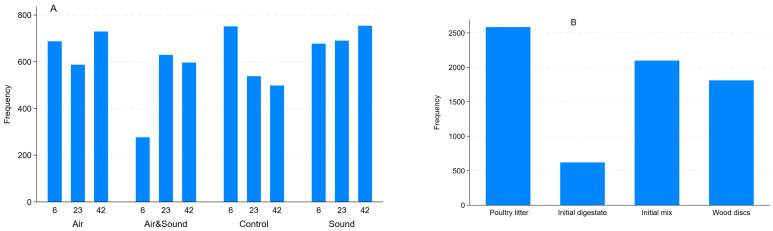

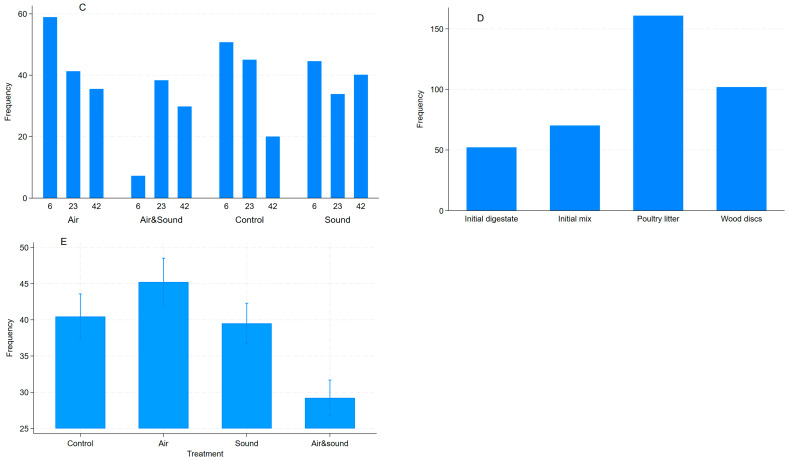
Frequency of virulence factor genes detected in the metagenomes by anaerobic digester treatment group (**A**) and experimental component (**B**); mean read counts by treatment group (**C**), and experimental component (**D**); model predicted number of reads comparing the anaerobic digesters (**E**).

Across all metagenomes, 14,522 virulence factor gene (VFG) accessions were detected, of which 7412 (51%) and 7110 (49%) were detected from the AD treatment groups, the experimental components, and the wood discs, respectively. The total number of VFGs tended to decline in the control digesters while showing a tendency to increase over the sampling weeks in the AD groups that were micro-aerated, sound-treated, or both (Figure 7A). The highest number (i.e., diversity) of VFGs was detected in the metagenomes of the raw poultry litter fed to the ADs, which seemed to mostly have contributed to the initial mix, since the digestate seed had the lowest number of VFGs detected. It is also apparent that wood discs adsorb the VFGs (Figure 7B).

#### 2.3.1. Total Abundance of Virulence Factor Genes

The frequency of detection of VFGs decreased in the control digester; while it generally increased in the groups that were micro-aerated, sound treated, or both during the digestion process (Figure 7A). The PL feed had the highest number of VFGs detected, and the digestate seed had the lowest number of genes (Figure 7B). The mean read counts decreased in the control, micro-aerated, and sound-treated digesters while increasing in the digester that was treated with both micro-aeration and sound (Figure 7C). The poultry litter feed had the highest mean read counts followed by wood discs (Figure 7D). Micro-aeration significantly (*p* = 0.04) increased the mean read counts compared to the control digester with no significant (*p* = 0.656) effect of sound treatment. On the other hand, their combination significantly (*p* < 0.001) reduced the mean read counts when compared to the control digester (Figure 7E). This finding, once again, indicates the antagonistic effect of sound treatment when combined with micro-aeration, suggesting that this combined treatment can be a good mitigation strategy to reduce VFGs in animal manure.

#### 2.3.2. Origin Species by Genera Associated with Virulence Factor Genes

The VFGs were associated with 41 genera among the metagenomes of the experimental components and the wood discs; the digestate had the lowest number (n = 33) of genera associated with the VFGs, and the PL and the wood discs had the highest (36 genera each). Among the metagenomes of the AD treatment groups, VFGs were associated with 37 genera with the lowest number of genera being detected in the control AD; the micro-aerated, and sound-treated AD groups had 35 genera each. *Mycobacterium* and *Brucella* were the top two genera with the highest VFG accessions (Table 2 and Table 3).

Among the *Mycobacterium* genus, the highest number of VFGs was associated with *M. tuberculosis*. Poultry litter had the highest number (48%, n = 1526) of *M. tuberculosis*-associated VFGs followed by the wood discs (27%). While micro-aeration (28.3%, n = 1466) and sound treatment (31.7%) alone seem to increase *M. tuberculosis*-associated VFGs, their combination (18%) tends to decrease them relative to that of the control digester (22%). There seems to be an antagonistic effect whereby the effect of micro-aeration was countered by simultaneous sound treatment. While the number of *M. tuberculosis*-associated VFGs initially declined and then increased in control and micro-aerated digesters, they consistently increased over the sampling weeks in the sound and air-sound-treated digesters.

*Brucella melitensis*, *B. suis*, and *B. abortus* were the three species with the VFGs detected in all metagenomes. Micro-aeration and sound alone tend to increase *Brucella*-associated VFGs, while their combination tends to decrease the number of VFGs detected relative to the control digesters.

#### 2.3.3. Origin Species Found in the Taxa Profiling of the Present Study

Only 3.4% (486/14,522) of VFG accessions had the origin species found in the taxa profiling of the current study. Strikingly, the vast majority (83%, n = 486) of these were detected in the initial mix, which were remotely followed by PL (15%). The species found in the current taxa profiling were unidentified *Enterococcus* species in the micro-aerated and sound treated (2), control (1), and sound (1) ADs. *Pseudomonas aeruginosa* (98.8%, 399/404) and *Acinetobacter* species (1.2%, 5/404) were detected in the initial mix. From the PL, *Enterococcus faecalis* (18.9%, 14/74) and *Escherichia coli* (78.4%, 58/74) were found in the taxa profiling of the current study.

## 3. Discussion

To reiterate, our main objective was to investigate the effect of the AD of poultry litter on antimicrobial resistance genes and see if the effect would be improved by micro-aeration and/or sound treatments. We used shotgun metagenomic sequencing of the total DNA as opposed to previous studies that heavily relied on PCR and to a lesser degree based on bacterial culture. Shotgun sequencing of the total DNA provides a comprehensive understanding of the microbiome, resistome, and virulence factor genes [20]. A study that specifically focused on the effect of AD on the total microbiome was previously published [19]. Here, we report the effects of AD with or without micro-aeration and/or sound treatments primarily on the resistome and secondarily on the virulence factor genes.

The fact that the total number of ARGs detected declined in the control digester, while they increased over time in AD groups treated with micro-aeration and/or sound, with a higher synergetic effect of their combination by greatly increasing the number of ARGs detected, shows that the AD system, especially if supplemented with little air and/or sound, enriches ARGs. This effect could be attributed to the increased microbial activity promoted by micro-aeration and sound [19]. Thus, AD can act as a hotspot for ARGs mainly through horizontal gene transfer among the microbial population [21,22]. Although this finding was not the desired outcome, it is consistent with our previous studies [8,14,15]. The effect of AD with respect to ARGs should be further evaluated at different temperatures, since the present study was conducted at ambient temperatures. Temperature is an important parameter that affects the effectiveness of AD, and it was shown that thermophilic AD processes are more effective at reducing ARGs from animal manure [3,8,23]. Agga et al. [8] extensively reviewed the general utilization of anaerobic digestion in various animal species and its specific effect on the removal of tetracycline resistance genes. It was shown that anaerobic digestion is widely used for the treatment of dairy manure and less frequently for swine manure. Anaerobic digestion was used scarcely for the management of poultry litter, which is the focus of the current study.

Another unique aspect of this study was the analysis of the individual components that were added into the digesters, which would provide information regarding the potential source of the ARGs into the system. In this regard, the PL had fewer number of ARGs detected than the initial digestate and the initial mixes, suggesting that the initial digestate contributed more to the initial mixture and/or that the mixing process increased the likelihood of detecting the ARGs by acting as a constant mixer [24]. Furthermore, the wood discs had the highest number of ARGs detected, presumably because they provided a larger surface area for the adsorption of the genes. Since the wood discs were added to the AD processes to mimic the bedding material in the broiler houses, our finding indicates the importance of recalcitrant bedding materials to adsorb ARGs during the anaerobic digestion of poultry litter. Since both the raw stored PL used for the AD experiment and the initial digestate used to seed the ADs were obtained from the same broiler farm, the presence of ARGs in the PL may indicate the antimicrobial-resistant bacterial burden in the chicken and that the digestate from a commercial AD system still contains ARGs that may spread through its use as fertilizer [22].

The four most abundant (and prevalent) antimicrobial resistance classes in the present study were MLS, tetracyclines, aminoglycosides, and glycopeptides. Similarly, the ARGs detected from the PL previously obtained from the same farm and used as a soil amendment in field experiment were dominated by MLS, which was followed to a lesser extent by tetracyclines and aminoglycosides [7]. Although on-farm studies are required, our findings indicate that resistance to these antimicrobial classes may be widespread on the broiler farm, which may spread into the environment through the land application of the digestates and the sludge. This is supported by the fact that the ARGs were not removed, and at times, they even were enriched during the anaerobic digestion of poultry litter in our experiment.

Knowing the mechanisms by which bacteria develop resistance to various antibiotics is important to understand the epidemiology of AMR and to develop mitigation strategies [11]. Three mechanisms of MLS resistance have been described [25,26]: (1) target modification mediated by rRNA *erm* methylases that alter a site in 23S rRNA common to the binding of macrolides, lincosamides, and streptogramin B antibiotics; (2) drug inactivation by enzymes (EreA and EreB) that hydrolyze the macrolides and phosphotransferases (*mph*A) that phosphorylate the drugs; and (3) efflux pumps (*msr*A, *msr*B, *mef*A, *mef*E). These three mechanisms were detected at similar frequencies in the present study, indicating the complexity of antimicrobial resistance [11]. The three main tetracycline resistance mechanisms including efflux, ribosomal protection, and drug inactivation described in the literature [27] were also detected in the current study.

The *van*R and *van*S genes were the dominant glycopeptide resistance genes, which are a canonical two-component regulation system that is required for vancomycin resistance [28]. The most prevalent sulfonamide resistance gene detected among all samples, *sul*1, is regularly tracked as a surrogate marker for the dissemination of ARGs and anthropogenic influence on the environment, such as in wastewater treatment and the decontamination of ARGs [29]. The two fosfomycin resistance gene families detected were plasmid-mediated *fos* and *fom* genes that inactivate the fosfomycin antibiotics [30]. The higher prevalence of the fosfomycin resistance genes in the untreated PL samples may indicate that poultry litter was the source, and these genes may be prevalent in the broiler production. As such, PL testing can be proposed to monitor AMR in broiler production.

Resistance to phenicols is conferred through three mechanisms: chloramphenicol O-acetyltransferase (CAT), resistance due to specific exporters (efflux), and the MDR transporter system [27]. Genes belonging to two mechanisms, CAT and efflux, were detected in the present study. While the phenicol resistance genes were enriched during anaerobic digestion of the poultry litter, the digestate samples were dominated (>90%) by the CAT-encoding genes. The finding clearly shows the shift in the underlying microbial population toward ones carrying the CAT genes. The nonspecific multidrug resistance genes detected were mainly the *oqx* group. OqxAB is a multidrug efflux pump that confers resistance to a wide range of antibiotics and biocides [31]. The higher frequency of detection of the MDR genes in the micro-aerated and sound-treated AD groups than in the PL feed and micro-aeration and sound combination-treated AD groups shows the enrichment of these genes during mixing and during anaerobic digestion processes when supplemented with micro-aeration and sound independently. Furthermore, combination treatment with micro-aeration and sound had antagonistic effects in reducing these genes, which may be considered as a potential mitigation strategy.

Biocides, especially QACs, are the commonly used disinfectants in the broiler production to control bacterial pathogens [32]. Often, the genes conferring resistance to QACs are associated with multidrug resistance in bacterial pathogens [33]. The lower detection of the *qac* genes in the digestate samples, regardless of treatment group, than the initial PL and the digester mixes shows that while conventional AD or its modifications with micro-aeration and/or sound treatments reduce the total number of biocide resistance genes such as the *qac* genes, they could not completely remove them.

The combination of micro-aeration and sound treatment had an antagonistic effect on the abundance of virulence factor genes during the anaerobic digestion of poultry litter. While micro-aeration treatment alone increased the mean abundance of VFGs, sound treatment did not have a significant effect. However, when sound was complemented with micro-aeration, it counteracted its effect, whereby the combination treatment reduced the mean abundance of the VFGs. Combining micro-aeration and sound treatment during anaerobic digestion can be explored as a mitigation strategy to reduce virulence factor genes for the safe utilization of animal manure. The initial digester mix had the highest number of *Brucella*-associated VFGs followed by wood discs and the PL. This finding suggests that mixing increased the likelihood of detecting these genes than the individual components. The bacterial species found in the current taxa profiling are common fecal bacteria, which aligns with the fact that the digested PL also consists of fecal matter in addition to bedding material.

## 4. Materials and Methods

### 4.1. Digester Design and Sampling

Digester design and sampling descriptions can be found elsewhere [19]. Briefly, the experiment was conducted in four digesters constructed from 208 L polyethylene tanks fitted with waste inlets and outlets. The digesters were maintained at ambient temperature. The digester experiment consisted of four treatments: control (CON), micro-aeration treated (MAT), sound-treated (SND), and micro-aeration and sound-treated (MST). For the micro-aerated tanks, 200 mL of air was supplied to the sludge layer using H-manifold for 15 min in four equally spaced intervals over 24 h. For the sound-treated tanks, one waterproof 4 inch 2-way speaker rated at 120 W was placed above the sludge layer. Amplification with a 1 kHz sine wave was provided with an amplifier rated at 20 W with gain set to half volume and operated continuously. The 1 kHz sine wave was chosen because in previous research, a 1 kHz sine wave was found to produce high-intensity bubble harmonics in wastewater that presumably extended into ultrasonic frequencies [18]. Control digesters did not receive any micro-aeration or sound treatment. The physicochemical parameters of the digesters were previously reported [18,19].

The digesters were initially seeded with 20 L of digestate obtained from a commercial mesophilic digester operated approximately at 43 °C. The digesters were fed every week with poultry litter (PL) obtained from a large (>500,000 birds per year) commercial broiler chicken farm in Kentucky, which is typical of the U.S. poultry production system. For the startup phase, the four digesters were fed 400 g PL in 4 L of water each week and operated similarly for the first six weeks. After the startup phase, sound and micro-aeration treatments were applied, and the amount of PL fed to the digesters was gradually increased every six weeks. Seven wood disks cut from tulip poplar boards (*Liriodendron tulipfera*) were placed in each digester at the beginning of the experiment, since the producer stated that the poultry bedding was composed of either *Pinus* spp. or poplar chips.

Digestate samples were collected at week 6, corresponding to the end of the startup phase during which all digesters were similarly fed 400 g PL per week with no sound or micro-aeration treatments initiated; at week 23, when the digesters had been fed 600 g PL per week for the previous 6 weeks; and at week 42, at which point the digesters had been fed 1 kg PL per week for 4 weeks and 800 g PL per week for the previous two weeks. In addition, we sampled the digestate obtained from the commercial anaerobic digester to seed the digesters, an initial mixture of PL and digestate seed at week 0 as baseline values. Raw poultry litter fed to the digesters were collected on weeks 0, 6, 23, and 42. Finally, swipes from the wood disks were obtained using sterile cotton swabs at the end of the anaerobic digestion experiment. Overall, 21 samples consisting of digestate samples obtained at three time points (weeks 6, 23 and 42), selected based on the feeding schemes of the digesters with varying amounts of PL, from CON (FAESRU.JL. 1–3), SND (FAESRU.JL. 4–6), MST (FAESRU.JL. 7–9) and MAT (FAESRU.JL. 10–12) digesters, and raw PL feeds (FAESRU.JL. 13–15, 19), wood disks (FAESRU.JL. 16–17), initial digestate seed (FAESRU.JL. 18), and initial week 0 mix (FAESRU.JL. 20–21), were collected. The samples were stored at −20 °C until processed.

### 4.2. DNA Extraction, Library Preparation, Sequencing and Bioinformatics Analysis

Samples were processed and analyzed with the shotgun metagenomic sequencing service (Zymo Research, Irvine, CA, USA). DNA extraction, library preparation and sequencing protocols were previously described [19]. Briefly, DNA was extracted using ZymoBIOMICS^®^-96 MagBead DNA kit (Zymo Research, Irvine, CA, USA) according to the manufacturer’s instructions. Genomic DNA samples were profiled with shotgun metagenomic sequencing. Sequencing libraries were prepared with an Illumina^®^ DNA library preparation kit (Illumina, San Diego, CA, USA) with up to 500 ng DNA input following the manufacturer’s protocol using unique dual-index 10 bp barcodes with Nextera^®^ adapters (Illumina, San Diego, CA, USA). All libraries were pooled in equal abundance, which will result in sufficient normalization and a similar number of reads per sample. The final pool was quantified using qPCR and TapeStation^®^ (Agilent Technologies, Santa Clara, CA). The final library was sequenced on the NovaSeq^®^ (Illumina, San Diego, CA, USA) platform. The ZymoBIOMICS^®^ microbial community standard (Zymo Research, Irvine, CA, USA) was used as positive controls for each DNA extraction and library preparation. Negative controls (i.e., blank extraction control, blank library preparation control) were included to assess the level of bioburden that may be carried by the wet-lab process.

Raw sequence reads were trimmed to remove low-quality fractions and adapters with Trimmomatic-0.33 [34] quality trimming by a sliding window with a 6 bp window size and a quality cutoff of 20, and reads smaller than 70 bp were removed. Antimicrobial resistance and virulence factor gene identification was performed with the DIAMOND sequence aligner [35]. The data of aligned antimicrobial resistance gene sequence reads were classified by gene (i.e., the sequence accession), group (gene-level group for that sequence, e.g., *tet*(W)), resistance mechanism (the biological mechanism of resistance, e.g., efflux), antimicrobial class (e.g., MLS, aminoglycosides) and type (antibiotics, biocides, multi-drug resistance).

### 4.3. Statistical Analysis

The richness of ARGs was defined as the observed number of unique ARGs (gene group) present in a sample and was estimated separately for each antibiotic, biocide, and multi-drug resistome types as well as aggregated at the mechanism and antimicrobial class levels. The resistome composition at the ARG level, class and mechanisms, and virulence factor genes were visualized using bar plots in STATA version 19.5 (StataCorp LLC, College Station, TX, USA). Resistance genes were generally grouped into four general mechanisms: target alteration (modification, protection), drug inactivation, decreased permeability, and increased efflux [36]. Negative binomial regression was used to compare the read counts of ARGs and VFGs aggregated by the treatment groups against the control.

## 5. Conclusions

On-farm anaerobic digesters maintained at ambient temperature are not effective for the complete removal of antimicrobial resistance and virulence factor genes from poultry litter. First, micro-aeration and the sound treatment of anaerobic digesters, although effective to increase the biogas production, which is a primary goal of the anaerobic digestion of animal manure, tend to enrich antimicrobial resistance genes and virulence factor genes. Second, similar antimicrobial resistance gene counts were found between the raw poultry litter and the digestates regardless of the treatment group, indicating that anaerobic digestion was not effective in removing antimicrobial resistance genes. Third, the bedding materials such as wood discs commonly used in commercial broiler production can adsorb antimicrobial resistance genes and the virulence factor genes. Nevertheless, the antagonistic effect of sound treatment when combined with micro-aeration countering the effect of micro-aeration alone was observed in reducing virulence factor genes. Thus, the combination treatment could be explored further as an effective strategy in reducing the burden of virulence factor genes in the anaerobic digestion of poultry litter. Overall, our study clearly shows the need for post-anaerobic digestion treatments such as composting to further reduce the risk of dissemination of antimicrobial resistance genes and bacterial virulence factor genes through poultry litter land applications.

**Implications, contributions, and future research:** No published studies were found that explicitly applied shotgun metagenomic sequencing to anaerobically digested poultry litter digestate with a direct analysis of ARGs and virulence factor genes, much less the novel application of micro-aeration, sound, or their combination treatment. Most existing studies used qPCR-based approaches, standard metagenomic microbial profiling, or focused on non-AD conditions. The shotgun metagenomic sequencing of the digestate could comprehensively unravel the fate of ARGs, virulence factor genes, and mobile genetic elements (MGEs), and link them to specific bacterial hosts. Existing non-AD manure metagenomes highlight a high baseline of ARGs and virulence factor genes—necessitating high-resolution AD studies to assess treatment effectiveness. In a laboratory batch experiment, Atanasova et al. (2025) [37] performed a high-throughput qPCR analysis (not shotgun), tracking 374 ARGs in poultry litter and AD digestate. It was found that although AD reduced the total ARG levels, it increased the diversity of ARGs detected, and the temporal changes in the digestate samples were minimal. While not shotgun-based, this study supports our finding that ARGs persist in post-AD. Similar to our study, that study also found ARGs conferring resistance to aminoglycosides, MLS, glycopeptides, and tetracycline as dominant antimicrobial classes.

In this regard, the current study is the first report to comprehensively evaluate the effectiveness of anerobic digestion of animal manure and poultry litter on antimicrobial resistance and bacterial virulence factor genes. Since the present study is unique and novel, our findings can serve as a foundation for future research. First, unlike previous studies, we used metagenomic sequencing to be able to evaluate treatments on the entire resistome rather than targeting only a few specific genes, which is the inherent limitation of PCR-based approaches. Second, in addition to traditional anaerobic digestion, we also evaluated the novel approaches of adding little air and sound to the system. The fact that sound reduced the abundances of ARGs and VFGs is promising and should be further investigated in large-scale field studies. Third, while previous studies were mainly laboratory-based experiments, the current study was conducted under field conditions, which provides real-life evidence that is applicable to anaerobic digesters maintained by farmers [8].

Although there are some limitations, the current study showed that anaerobic digestion, while suitable for biogas production from biological waste, is not an effective animal manure management technology for the complete removal of ARGs and VFGs from animal manure and poultry litter. Nevertheless, it is worth mentioning some limitations of the current study that can be addressed in future studies. The present study evaluated the effectiveness of anaerobic digestion at ambient temperature to mimic a cost-effective approach that can be maintained by farmers. As previously reviewed [8], thermophilic anaerobic digesters were shown to be more effective in experimental studies; however, they are expensive, and high temperature may also affect the performance of anaerobic digestion to produce biogas by disrupting the microbial activities. The main limitation of the current study was its small sample size, which was due to operational and economic constraints; this has resulted in a decreased statistical power to observe the measurable effects of anaerobic digestion at the treatment level. Due to the lack of biological replicates, we could not analyze the data over time, and our inference was merely based on observed values. Multi-center studies operating under the same conditions would alleviate this issue. Alternatively, and more applicably, a longitudinal study could be conducted with an adequate cohort size of commercial broiler farms who operate anaerobic digesters for poultry litter management.

## Figures and Tables

**Table 1:** The number of antimicrobial resistance gene accessions detected by antimicrobial class categorized by treatment groups and experimental components.

Antimicrobial Class	Anaerobic Digester Group				
	Control	Air	Sound	Air & Sound	Total
Aminoglycosides	90	83	87	77	337
Beta-lactams	14	12	19	8	53
Fluoroquinolones	1	2	1	0	4
Folate pathway inhibitors	25	15	21	16	77
Fosfomycin	5	8	8	7	28
Glycopeptides	53	61	53	48	215
MLS	140	138	152	129	559
Multidrug resistance	6	8	9	3	26
Mupirocin	3	4	5	1	13
Phenicols	12	14	11	11	48
QACs	5	2	5	2	14
Rifamycins	11	15	14	8	48
Tetracyclines	135	125	139	119	518
Thiopeptide	3	3	3	1	10
Total	503	490	527	430	1950
**Antimicrobial Class**	**Experimental Components**				
	**Initial Digestate**	**Poultry Litter**	**Initial Mix**	**Wood Disc**	**Total**
Aminoglycosides	16	84	55	50	205
Beta-lactams	2	5	44	10	61
Fluoroquinolones	1	3	4	0	8
Folate pathway inhibitors	5	35	7	18	65
Fosfomycin	3	25	1	8	37
Fusidic acid	0	3	0	0	3
Glycopeptides	20	18	8	25	71
MLS	40	166	53	97	356
Multidrug resistance	2	3	10	4	19
Mupirocin	1	6	3	3	13
Phenicols	4	3	16	12	35
Polymyxins	0	0	1	0	1
QACs	1	8	17	5	31
Rifamycins	4	17	9	13	43
Tetracyclines	37	106	59	82	284
Thiopeptide	0	6	0	2	8
Total	136	488	287	329	1240

MLS: macrolide–lincosamide–streptogramin resistance; QACs: quaternary ammonium compounds.

**Table 2:** Virulence factor gene accessions detected from the metagenomes of the experimental components with their associated genera.

Origin Gena	Initial Digestate	Initial Mix	Poultry Litter	Wood Discs	Total
*Acinetobacter*	1	5	0	0	6
*Actinobacillus*	12	37	41	31	121
*Aeromonas*	0	1	0	0	1
*Aspergillus*	0	2	0	4	6
*Bacillus*	4	7	24	21	56
*Bartonella*	2	13	5	10	30
*Bordetella*	6	24	8	28	66
*Borrelia*	0	0	2	1	3
*Brucella*	150	487	317	382	1336
*Burkholderia*	15	74	45	51	185
*Campylobacter*	4	14	13	40	71
*Candida*	3	7	0	3	13
*Chlamydia*	2	2	4	4	12
*Clostridium*	1	1	10	2	14
*Coccidioides*	1	2	3	2	8
*Corynebacterium*	0	0	1	0	1
*Coxiella*	1	2	3	2	8
*Cryptococcus*	4	13	19	18	54
*Enterococcus*	10	12	57	15	94
*Escherichia*	21	59	95	67	242
*Francisella*	24	87	83	51	245
*Haemophilus*	10	25	29	20	84
*Helicobacter*	7	21	22	25	75
*Legionella*	9	34	21	22	86
*Leishmania*	1	0	1	2	4
*Listeria*	19	34	122	52	227
*Mycobacterium*	127	300	805	461	1693
*Neisseria*	16	54	58	39	167
*Paenibacillus*	1	0	2	0	3
*Pasteurella*	0	8	2	1	11
*Pseudomonas*	28	403	88	81	600
*Saccharomyces*	1	0	4	2	7
*Salmonella*	30	76	105	82	293
*Shigella*	5	53	31	35	124
*Staphylococcus*	18	23	138	44	223
*Streptococcus*	51	74	302	114	541
*Toxoplasma*	0	3	3	3	9
*Vaccinia*	0	2	0	0	2
*Vibrio*	21	81	71	49	222
*Victors*	0	0	1	2	3
*Yersinia*	14	57	49	44	164
Total accessions	619	2097	2584	1810	7110
Number of genera	33	35	36	36	41

**Table 3:** Virulence factor gene accessions detected from the metagenomes of anaerobic digester treatment groups with their associated genera.

Origin Genus	Air	Air &Sound	Control	Sound	Total
*Actinobacillus*	34	25	39	34	132
*Aeromonas*	0	1	0	0	1
*Bacillus*	14	10	11	14	49
*Bartonella*	7	9	5	7	28
*Bordetella*	21	17	21	25	84
*Borrelia*	3	2	2	2	9
*Brucella*	421	310	350	437	1518
*Burkholderia*	48	39	45	48	180
*Campylobacter*	28	25	18	24	95
*Candida*	4	2	3	3	12
*Chlamydia*	6	4	5	5	20
*Clostridium*	5	2	2	4	13
*Coccidioides*	1	0	1	1	3
*Corynebacterium*	0	0	0	1	1
*Coxiella*	3	3	2	3	11
*Cryptococcus*	10	8	7	9	34
*Enterococcus*	33	27	32	33	125
*Escherichia*	82	66	89	94	331
*Francisella*	71	65	74	74	284
*Haemophilus*	24	24	22	25	95
*Helicobacter*	26	17	17	25	85
*Legionella*	37	34	29	32	132
*Leishmania*	3	3	3	3	12
*Listeria*	76	60	72	75	283
*Mycobacterium*	459	290	345	511	1605
*Neisseria*	50	34	45	48	177
*Paenibacillus*	2	0	0	0	2
*Pasteurella*	1	1	0	1	3
*Pseudomonas*	69	45	59	84	257
*Saccharomyces*	1	0	0	1	2
*Salmonella*	99	83	120	114	416
*Shigella*	26	23	34	33	116
*Staphylococcus*	50	47	53	57	207
*Streptococcus*	176	135	163	174	648
*Toxoplasma*	3	2	3	2	10
*Vibrio*	**65**	**48**	**63**	**67**	**243**
*Yersinia*	45	40	53	51	189
Total accessions	2003	1501	1787	2121	7412
Number of genera	35	33	32	35	37

## Data Availability

Sequence data were deposited into the National Center for Biotechnology Information (NCBI) under Sequence Read Archive (SRA) and can be accessed by the BioProject ID: PRJNA1013451 with the following BioSample Accession numbers: SAMN37298551, SAMN37298552, SAMN37298553, SAMN37298554, SAMN37298555, SAMN37298556, SAMN37298557, SAMN37298558, SAMN37298559, SAMN37298560, SAMN37298561, SAMN37298562, SAMN37298563, SAMN37298564, SAMN37298565, SAMN37298566, SAMN37298567, SAMN37298568, SAMN37298569, SAMN37298570, and SAMN37298571.

## Supplementary Materials

The following supporting information can be downloaded at https://www.mdpi.com/article/10.3390/antibiotics15020153/s1, Table S1: Full resistome data. Table S2: Bacterial origin species for the alignments of antimicrobial resistance genes. Table S3: Frequency of all antimicrobial resistance genes detected and aggregated by antimicrobial classes and resistance mechanisms. Table S4: Frequency of all antimicrobial resistance genes tabulated by antimicrobial classes.
